# In Vivo Click Chemistry Enables Multiplexed Intravital Microscopy

**DOI:** 10.1002/advs.202200064

**Published:** 2022-06-24

**Authors:** Jina Ko, Kilean Lucas, Rainer Kohler, Elias A. Halabi, Martin Wilkovitsch, Jonathan C. T. Carlson, Ralph Weissleder

**Affiliations:** ^1^ Center for Systems Biology Massachusetts General Hospital 185 Cambridge St, CPZN 5206 Boston MA 02114 USA; ^2^ Department of Medicine Massachusetts General Hospital Harvard Medical School Boston MA 02114 USA; ^3^ Department of Systems Biology Harvard Medical School 200 Longwood Ave Boston MA 02115 USA

**Keywords:** cancer, click chemistry, drug delivery, intravital microscopy

## Abstract

The ability to observe cells in live organisms is essential for understanding their function in complex in vivo milieus. A major challenge today has been the limited ability to perform higher multiplexing beyond four to six colors to define cell subtypes in vivo. Here, a click chemistry‐based strategy is presented for higher multiplexed in vivo imaging in mouse models. The method uses a scission‐accelerated fluorophore exchange (SAFE), which exploits a highly efficient bioorthogonal mechanism to completely remove fluorescent signal from antibody‐labeled cells in vivo. It is shown that the SAFE‐intravital microscopy imaging method allows 1) in vivo staining of specific cell types in dorsal and cranial window chambers of mice, 2) complete un‐staining in minutes, 3) in vivo click chemistries at lower (µm) and thus non‐toxic concentrations, and 4) the ability to perform in vivo cyclic imaging. The potential utility of the method is demonstrated by 12 color imaging of immune cells in live mice.

## Graphical abstract

A click chemistry‐based technology that enables multiplexed in vivo imaging in mouse models is presented. This method uses a scission‐accelerated fluorophore exchange, which completely removes fluorescent signal from antibody‐labeled cells in vivo, allowing cyclic imaging for high multiplexing.

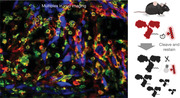

## 1 Introduction

The technical capabilities of fluorescence imaging have dramatically advanced over the last few decades and now include technologies with different spatial resolutions and imaging depths.^[^ ^1^ ^]^ Intravital microscopy (IVM) in particular^[^ ^2^, ^3^, ^4^, ^5^ ^]^ has been gaining interest as it allows dynamic serial imaging of specific cells,^[^ ^6^ ^]^ biological processes, and drug action^[^ ^7^, ^8^ ^]^ in an intact microenvironment. Borrowing concepts from IVM, clinical counterparts such as fluorescence guided surgery,^[^ ^9^ ^]^ dermoscopy,^[^ ^10^ ^]^ and confocal endomicroscopy^[^ ^11^ ^]^ have also been developed in the hope to ultimately perform molecular analyses in vivo (“optical biopsies”).^[^ ^12^ ^]^ Current fluorescence IVM approaches typically harness the entire optical spectrum and combined with fluorescent reporters allow imaging of approximately four to six targets in vivo before spectral overlap becomes a technical limitation.

High multiplexed immunofluorescence technologies (CYCIF, MIF, FAST) have been developed for tissue sections to interrogate biological complexity and heterogeneity.^[^ ^13^, ^14^, ^15^, ^16^, ^17^, ^18^ ^]^ Collectively, these methods and single cell RNA sequencing (scRNAseq) have unraveled a rich in vivo environment that is difficult to analyze serially in vivo without having high multiplexing capabilities. The spectral limitations of current IVM techniques thus hinder a broader functional analysis of different cellular players.

We hypothesized that new bioorthogonal chemistries could be used to achieve longitudinal multiplexing during IVM in live animals. A recently described bioorthogonal method (FAST)^[^ ^15^ ^]^ involved the creation of antibody‐*trans*‐cyclooctene (TCO)/linker‐fluorochrome conjugates where the construct could be quenched by the addition of a tetrazine black hole quencher (Tz‐BHQ). Unfortunately, the kinetics and concentration requirements of quenchers were not compatible with live cell imaging and quencher degradation products increased background signals. More recently, we have developed a scission‐accelerated fluorophore exchange (SAFE) method, which leverages cooperative bioorthogonal mechanisms to first quench and then completely remove fluorochromes from antibody‐labeled cells.^[^ ^19^ ^]^ The method reaches >99% quenching in seconds at low µm non‐toxic concentrations. We further optimized this method by reducing the Tz‐BHQ to simple Tz scissors more suitable for in vivo imaging. Here we show that this SAFE‐IVM allows repeat cyclic imaging (12 channels) of immune cells using dorsal window chambers (DWCs) in live mice, increasing the multiplexing capabilities that can be achieved in vivo.

## 2 Results

### 2.1 SAFE‐IVM Allows Rapid Kinetic Scission

**Figure 1:**
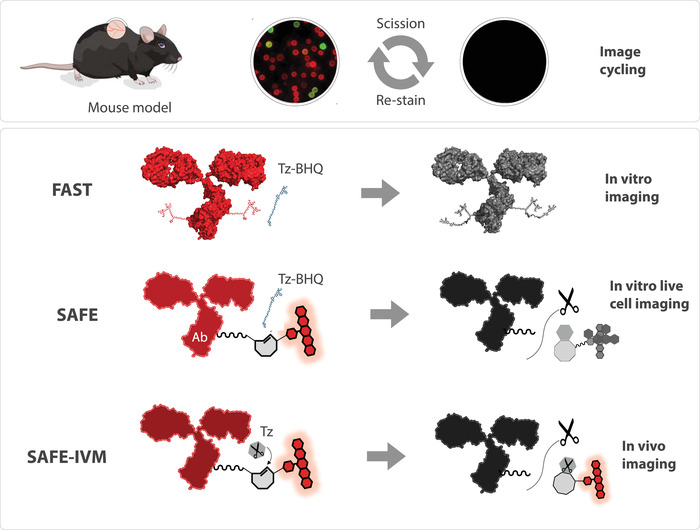
Conceptual cycling strategies for in vivo imaging. Intravital microscopy allows cellular imaging in murine window chambers, often used in transgenic mice expressing fluorescent proteins. Click chemistries such as FAST and SAFE are being explored to expand the repertoire of imaging channels by allowing additional cycles of antibody staining followed by quenching and re‐staining. The FAST method developed for in vitro staining^[^ ^15^ ^]^ uses a Tz‐BHQ to quench an antibody bound fluorochrome. The SAFE‐IVM method used here for mouse imaging is based on Tz‐scissors to cleave a C_2_TCO‐fluorophore off the linker in immolative fashion. This allows intravital imaging by eliminating unwanted background signal from BHQ3 degradation products.

The ability to perform higher multiplexing in vivo could enhance our ability to understand complex dynamic processes in vivo. Up to now, the primary method for intravital imaging has been the use of engineered cells and mouse models expressing different fluorescent proteins.^[^ ^20^, ^21^, ^22^ ^]^ We reasoned that these highly successful methods could be further enhanced by in vivo click chemistries.^[^ ^23^, ^24^, ^25^ ^]^ The goal would be to stain tissue with fluorescently labeled antibodies complementary to fluorescent proteins and then quench the former followed by repeat cycles to stain for additional cell markers. One such approach (FAST) involves the use of tetrazine (Tz)/TCO chemistry for site‐specific delivery of fluorescence quenchers.^[^ ^15^ ^]^ This method showed surprisingly fast quenching reaction rates in vitro, three orders of magnitude faster (*t*_1/2_ < 1 s) than predicted. While the method is well suited for in vitro cycling, a signal rebound upon quencher degradation in longitudinal studies (at 37 °C) precludes its use in live cells. We therefore re‐designed the scaffold so that the use of a C_2_TCO^[^ ^26^ ^]^ would lead to immolation of the linker so that the quenched fluorochrome could be washed away. We termed this method SAFE and proceeded to synthesize a portfolio of C_2_TCO‐linked fluorophores (MB488, AF488, AF555, MB594, AF647) for antibody conjugation and multiplexed live cell/tissue immunofluorescence imaging.^[^ ^19^ ^]^ When we applied SAFE in vivo, however, we observed that the BHQ3 quencher can actually be internalized and degraded inside cells, causing the background fluorescence in the Cy3 channel to increase. Therefore, we modified the method and used scission only (without quenching) for in vivo imaging (SAFE‐IVM; **Figure** **1**).

**Figure 2:**
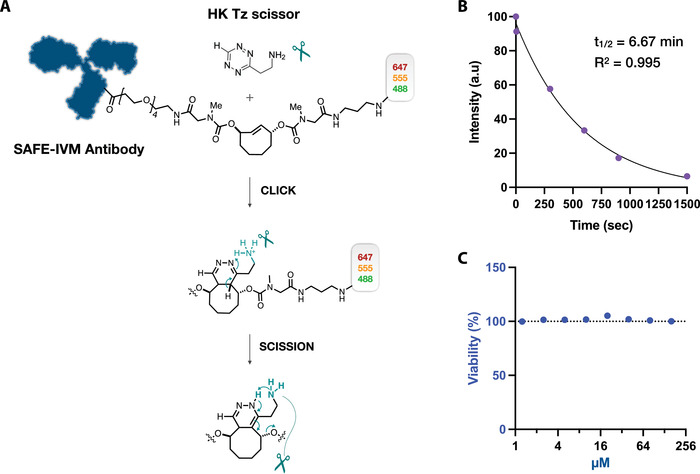
Synthesis, kinetics, and cell viability. A) The design of scission‐accelerated fluorophore exchange (SAFE) combines a click reaction with rapid fluorophore scission to remove the fluorophores from immunostained tissues. The H‐Tz modified with a NH_3_+ group (HK‐Tz) that enables complete cleavage (>99%) of C_2_TCO with fast click rates is used for fluorophore scission. HK‐Tz scissors click with antibody‐C_2_TCO‐fluorophore conjugates, followed by instantaneous tautomerization and scission of the fluorophores. The design of C_2_TCO itself ensures that the cleavage reaction proceeds irrespective of the Tz/TCO click orientation. B) Reaction kinetics in cells. A431 cells were stained with SAFE‐IVM labeled anti‐EGFR antibody and the fluorophores were released using HK‐Tz (40 µm). Incubation times with HK‐Tz include 0, 5, 300, 600, 900, and 1500 s. 95% of the signal was reduced in 30 min with a half life (*t*_1/2_) of 6.67 min. C) To test HK‐Tz toxicity, cell viability was measured at various micromolar concentrations (1.25, 2.5, 5, 10, 20, 40, 80, 160 µm). In this study, 40 µm HK‐Tz was used for cyclic imaging, which proved to be entirely non‐toxic to A431 cells. *N* = 6 replicates were measured.

The design of SAFE‐IVM exploits the ability of functionalized H‐Tetrazines (H‐Tz) to achieve fast and complete linker cleavage in reactions with C_2_TCO (**Figure** **2**A). H‐Tz modified with a NH_3_+ group (HK‐Tz) enabled complete cleavage (>99%) of C_2_TCO with fast click rates (390 m ^−1^ s^−1^); in solution, the results show that the click reaction reaches ≥99% completion within 2 min at concentrations of 10 µm C_2_TCO and ≈50 µm HK‐Tz.^[^ ^26^ ^]^ To quantify the combined click/scission reaction in cells, we synthesized a C_2_TCO‐AF647‐labeled anti‐EGFR antibody (SAFE‐IVM 647) which we used to stain live A431 cells. Incubation of cells with HK‐Tz (40 µm) showed that the cleavage was rapid (*t*_1/2_ = 6.6 min) and that >95% of the signal was gone after 30 min (Figure 2B). We also performed cell viability experiments at micromolar concentrations to assure that reactants were non‐toxic to cells. A431 cells were treated with HK‐Tz for 1 h and cell viability was measured 24 h later. Our data show that ≈99% of the cells remained viable up to 160 µm HK‐Tz, which was promising for live mouse experiments (Figure 2C). We have also tested the viability of cells in vivo by collecting a 1 h movie after three cycles of Hk‐Tz treatment and the cells were healthy and actively moving around (Movie S1, Supporting Information).

### 2.2 Staining and Scission Is Efficient In Vivo

**Figure 3:**
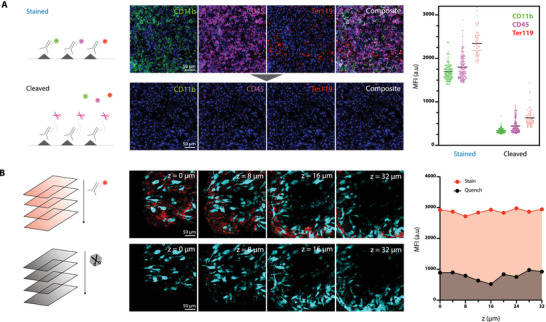
In vivo imaging with SAFE‐IVM chemistry. A) To determine the efficiency of scission‐accelerated fluorophore exchange in vivo, we labeled immune cells in a dorsal window chamber of a live B6 mouse using anti‐CD11b, CD45, and Ter119 SAFE antibody conjugates. Four channel imaging (green via anti‐CD11b‐MB488, magenta via anti‐CD45‐AF555, red via anti‐Ter119‐AF647 and blue Hoechst for nuclei). The in vivo imaging signal of labeled cells was between 1300 and 3200 RFU. 10 min after topical administration of Tz‐scissors the average signal had decreased to background fluorescence levels similar to baseline (grey area). B) The ability to stain and quench at different tissue levels was explored by z‐stack imaging from 0 to 32 µm depth. Shown are tumor cells in cyan (MC38‐BFP) and immune cells stained with anti‐CD45‐AF555 (red). Note that the quenching was similar at all depths investigated up to 32 µm.

We initially performed two key experiments in live mice to demonstrate that 1) fluorochrome scission is possible and sufficient to allow for cycling and 2) that the depth penetration of Tz scissors is sufficient for z‐stack imaging. For these experiments we used a DWC model in C57BL/6 mice in which MC38‐BFP tumor cells were implanted. We used three different antibodies targeting CD11b (anti‐CD11b‐MB488 targeting myeloid cells), CD45 (anti‐CD45‐AF555 targeting white blood cells), and Ter119 (anti‐Ter119‐AF647 targeting red blood cells) to measure fluorescence intensities before and after scission. Both staining and scission were performed in situ by directly applying reagents onto the tissue followed by washing. Care was taken to make sure that all reagents were at physiological pH (7.36) and correct osmolarities (300 mosm) to avoid adverse effects. As can be seen in **Figure** **3**A, scission was complete and a SNR of >4 was achieved for all three fluorochromes tested (MB488, AF555, AF647) in this study (Figure S1, Supporting Information). We also collected a time lapse video that shows temporal imaging of SAFE‐IVM during the staining and scission steps (Figure S2 and Movie S2, Supporting Information).

We next performed z‐stack imaging to determine the depth penetration of SAFE reagents following topical application. We chose topical rather than systemic applications, largely to preserve reagents. However, it is entirely possible to administer fluorescently labeled antibodies systemically prior to imaging.^[^ ^27^ ^]^ Given the importance of quantitating immune cells during tumor treatments, we chose CD45 to stain host cells in the tumor microenvironment for ≈5 min. After staining, cells were treated with HK Tz‐scissors for ≈15 min to remove fluorophores from antibody‐labeled cells. Our data show that leukocytes could be detected primarily inside and in the periphery of growing tumors up to tested depths of ≈30 µm (Figure 3B and Movie S3, Supporting Information, for additional targets). Following imaging, we administered the HK Tz‐scissors (MW < 200 Da) and observed consistent de‐staining of CD45 to the same depth of 30 µm. Collectively, the above results show that it is indeed feasible to perform in vivo labeling of specific cells and subsequent scission.

### 2.3 TME Immune Cell Profiling In Vivo

**Figure 4:**
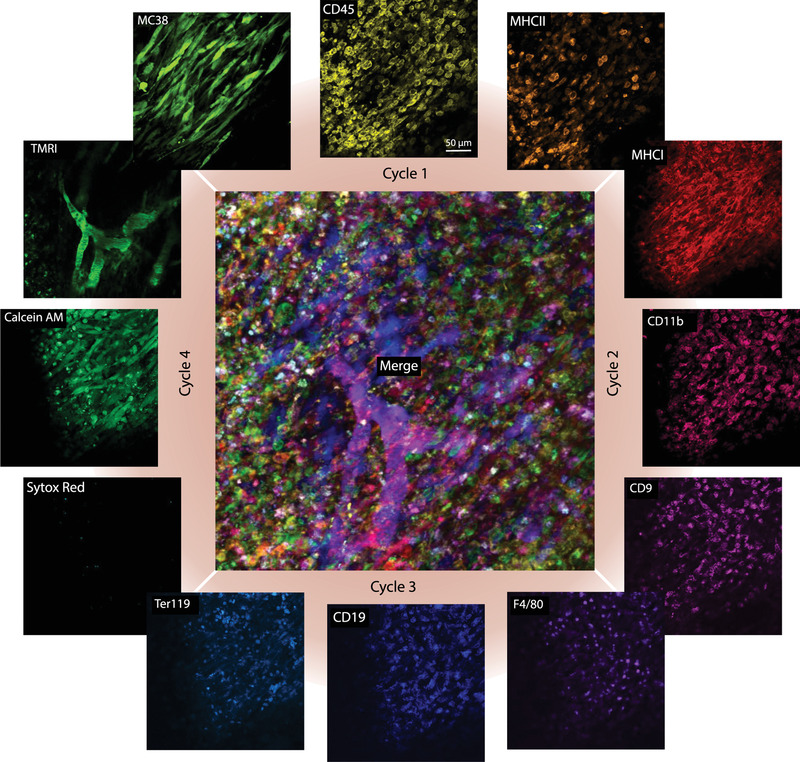
Multiplexed in vivo imaging. Immunocompetent C57BL/6 mice with a dorsal window chamber containing 7‐day old MC38‐BFP tumor cells (blue) were imaged for four different cycles of staining and quenching with different antibody conjugates. The 12 markers included Ly6G, MHCII, CD45 for cycle 1, CD11b, CD8, MHCI for cycle 2, CD18, F4/80, Ter119 for cycle 3, and Calcein AM, Sytox Red, TMRI for cycle 4 for visualization of live cells, dead cells, and vasculature, respectively. Note the complementary information obtained during each cycle of imaging. The vast majority of cells in cycle 4 are viable and the few observed SYTOX Red positive cells are expected in growing tumors.^[^ ^29^, ^30^ ^]^

Various immune cells play important roles in tumor progression and regression and have thus received growing interest in developing new therapeutic strategies.^[^ ^28^ ^]^ There are no single defining markers that can identify all subtypes of prognostically and therapeutically relevant immune cells and deep multiplexing has thus become the norm of analysis by cytometry. This approach unfortunately only yields “parts lists” without considering spatial or temporal information of motile cells. We thus decided to determine whether SAFE‐IVM of immunocytes is feasible. We developed and validated 12 SAFE‐IVM antibody conjugates for these proof‐of‐principle studies. Using the MC38‐BFP tumor window chamber model, we stained for all 12 markers within four cycles (total time < 2 h) (**Figure** **4**). The antibodies were separately tested using mouse splenocytes (Figure S3, Supporting Information) and validated in additional flow cytometry studies.^[^ ^15^, ^17^ ^]^ Each cycle was imaged for several minutes but this could be extended to track more motile cells (Figure S4, Supporting Information). To assess whether immunocytes were functional after four cycles of staining and scission, we performed live/dead cell staining after the last cycle. This Calcein AM staining showed that all cells were indeed viable.^[^ ^29^, ^30^ ^]^ Together these data show that it is feasible to perform multiple cycles of staining and scission in live mice.

### 2.4 In Vivo Deciphering of Nano Carrier Drug Distribution

**Figure 5:**
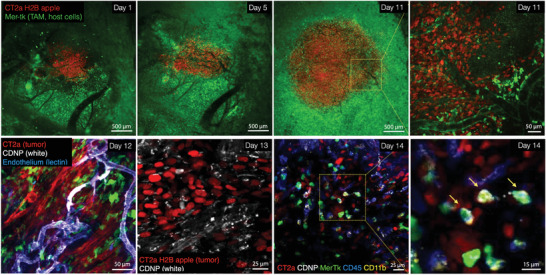
Serial imaging of glioblastoma in a brain window model during drug delivery. CT2a‐H2B‐mApple glioblastoma cells (red) were implanted intracranially in Mer‐Tk‐GFP mice (green host cells). Note the tumor growth and perivascular host cells infiltration on day 11. At that point, a pacific blue‐labeled cyclodextrin nanoconstruct (CDNP; white) was injected systemically and imaged over two days to determine its cellular distribution (pharmacokinetics). On Day 14, SAFE‐IVM was performed to 1) determine the feasibility in the presence of other fluorescent proteins and conjugates and 2) identify the specific cell types in which the nano construct resided. A total of two cycles were obtained with anti‐CD11b‐AF555 (cycle 1) and anti‐CD45‐AF647 (cycle 2). The CDNP (white) primarily accumulated in triple positive cells staining for CD11b (yellow), CD45 (blue), and Mer‐Tk (green), that is, myeloid‐derived tumor associated macrophages.

To further explore the in vivo click chemistry technique, we applied it to questions of glioblastoma (GBM) drug delivery, and particularly the cellular localization of an IL‐12 inducer.^[^ ^31^, ^32^ ^]^ The latter consisted of a 38 nm cross‐linked cyclodextrin nanoparticle (CDNP) that can be loaded with a cIAP inhibitor capable of inhibiting the non‐canonical NFkB pathway. This in turn leads to local IL‐12 upregulation in target cells with antineoplastic effects based on immune cell regulation^[^ ^27^, ^31^, ^32^ ^]^ as part of a larger effort to induce local^[^ ^33^ ^]^ rather than systemic IL‐12.^[^ ^34^ ^]^ Up to now, what was unknown in this paradigm was the specific cell type in which CDNPs localize in the GBM microenvironment. To study this effect we used a Mer‐TK mouse model^[^ ^35^ ^]^ in which all macrophages contain GFP and implanted CT2a tumor cells tagged with H2B‐apple in a cranial window chamber. Tumor growth was monitored by serial IVM imaging and CDNP drug distribution was imaged by Pacific Blue (PB) labeling of the nano carrier. We first saw the CDNP carrier in the vascular space with a half life of 62.5 ± 4.75 min.^[^ ^36^ ^]^ Within 24 h after administration, the CDNP was largely located in cells. To identify the specific cellular components at this point we performed SAFE‐IVM to additionally label cells for CD45 (all leukocytes) and CD11b (myeloid cells) expression in two cycles (**Figure** **5**) Our data show that the predominant fraction of CDNP resided in triple marker positive cells (CD45+, CD11b+, Mer‐TK+), that is, myeloid‐derived tumoral macrophages but not neutrophils or microglia (Figure S5, Supporting Information). This type of experiment could easily be expanded to other cell type markers relevant in GBM therapy. Overall this data confirms the ability to apply click chemistry in highly complex mouse models in an effort to decipher the kinetics and distribution patterns of drugs.

## 3 Discussion

Our results demonstrate that 1) cycling immunochemistry is technically feasible in live animals, 2) the ON/OFF signal dynamics before and after scission are sufficient to allow for repeat high quality image acquisition, and 3) the SAFE‐IVM method is non‐toxic and does not lead to cell death or inflammatory responses. The ability to perform multiplexed in vivo imaging could transform biological interrogation of diverse cell populations.

### 3.1 Technical Considerations

A key aspect to in vivo imaging is the spatial distribution and depth penetration of cyclic imaging agents (fluorochrome labeled antibodies and Tz‐scissors) during each cycle of imaging. While it is feasible to inject antibody conjugates systemically and then perform scission locally, the pharmacokinetics are generally not amenable to fast cycling. We therefore used local administration of both labeled antibodies and Tz‐scissors, followed by wash steps with saline. The advantage of this approach is rapid distribution and staining in horizontal *x*/*y* directions (i.e., perpendicular to the window chamber and optics) along the tissue surface but slower penetration vertically along the *z*‐axis (i.e., toward greater depths) into the tissue. Prior modeling had shown that antibodies with an effective diffusivity of 10 µm^2^ s^−1^ are expected to diffuse throughout ≈100 µm thick viable tumor tissue in 15 min, something to consider when there is a need to image deeper. The distribution of the Tz‐scissors is faster, given its much smaller molecular weight (<200 Da). Our experimental data shows that staining and scission can be performed in at least the superficial 30 µm during the cycles.

To enable deeper imaging one would have to use smaller affinity ligands rather than whole antibodies. Smaller affinity ligands for better tissue penetration include scFv fragments, camelid V_V_H (nanobodies),^[^ ^37^, ^38^ ^]^ or affinity peptides. Tissue penetration of drugs is well researched^[^ ^39^ ^]^ and has been modeled.^[^ ^40^, ^41^ ^]^ There is a body of literature connecting various parameters with permeability including extracellular matrix,^[^ ^39^ ^]^ cellularity,^[^ ^42^ ^]^ tumor type, perivascular macrophages,^[^ ^43^ ^]^ and therapeutic interventions.^[^ ^44^ ^]^ It is possible that modulation of these could result in further improved permeability. Finally, additional methods to improve depth penetration include the use of multiphoton rather than confocal imaging, tissue clearing,^[^ ^45^ ^]^ or optoacoustic imaging.^[^ ^46^, ^47^ ^]^

A second technical issue is the achievable SNR during imaging. Empirical data suggests that SNR levels of >4 are desirable to image objects reliably by IVM. The SNR is a complex function determined by quality of antibody (affinity, nonspecific binding), fluorochromes used (spectral window, brightness, quenchability by light) and importantly, fluorescence background levels (autofluorescence, contributions from fluorescent proteins if used, residual fluorescence from previous quenching cycles) during cycling. We found that scission was best at higher wavelengths but that SNR was roughly similar across the three channels (Figure S1, Supporting Information). We attribute the latter to the higher fluorescence in the green channel. Based on in vitro experiments, we expect that it is feasible to extend cycling to the far red wavelength by using additional far‐red dyes, for example, AF680, 700, 750, and 790 SAFE‐IVM probes. Together with the recent introduction of NIR capable IVMs and detector units, this would enable extended multiplexing with better tissue penetration and lower scattering.

A third technical consideration is the number of cycles that can be achieved in vivo. Prior experiments with fixed cells or frozen tissue have shown that it is possible to image at least for ten cycles. In live cells, we had shown that the SAFE method could be used for at least four cycles. In the current study of live tissue, we also imaged for up to four cycles. The SAFE‐IVM did not include black hole quencher 3 (BHQ3) that was used in previous studies to quench the signal from fluorophores.^[^ ^19^ ^]^ When BHQ3 was used for in vivo imaging, we observed that the quencher was internalized and degraded over time inside a subset of live cells, causing a background signal in the Cy3 channel. Due to this reason, we used Tz‐scissors to release the fluorophores instead of quenching them. We are currently developing Tz/TCO pairs that can both quench and comprehensively release the fluorophores to better increase de‐staining efficiency and we expect that using the new reagents, in vivo cycling could be further increased to at least ten cycles. One of the practical aspects with IVM however is the mobility of cells (neutrophils, macrophages, cancer cells) and importantly tissue movement due to cardiovascular, respiratory, or skeletal motion. In order to image successfully over multiple cycles, it is necessary to minimize any such motion through the judicious use of several compensation technologies.^[^ ^48^, ^49^, ^50^, ^51^ ^]^ In the current study, we used complete physiologic monitoring, immobilization of window chambers on the microscope stage, and image post processing to compensate for image distortions present during image acquisition.

### 3.2 SAFE and SAFE‐IVM Chemistry Is Safe

SAFE probes were designed to allow for extremely fast cycling in the span of a few seconds, while using micromolar concentrations of reagents for gentle quenching and irreversible fluorophore scission. The combination of ultrafast kinetics, very low concentrations, and general non‐toxicity of TCO/Tz reagents contributes to the very favorable safety profile. In prior experiments leading up to the current work we had not observed any cellular toxicity of reagents up to the µm level, which we corroborated for the HK‐Tz scissors here. We collected a 1 h time lapse video after multiple cyclings (*N* = 3) and showed that cells remain highly mobile and viable in vivo (Movie S1, Supporting Information). We have not been able to see any functional consequences in immune cell labeling on effector function in vitro. Furthermore, Tz containing drugs are in use clinically, for example, the Tz moiety has been used as a prodrug for a pyridazine,^[^ ^52^ ^]^ attesting to the general biocompatibility of the structural motif.

### 3.3 Use of SAFE‐IVM with Existing Imaging Methods

Conventional intravital confocal and multiphoton microscopy is commonly performed using a variety of fluorescent proteins in various configurations. An important aspect of this work was to test that SAFE‐IVM probes are compatible with and complementary to the information derived from 1) fluorescent proteins (e.g., GFP, RFP, YFP, BFP) commonly used in transgenic and tumor mouse models, 2) other small molecule fluorochromes (e.g., DAPI or Hoechst for nuclear stain; Calcein or SYTOX Red for viability stain), or 3) fluorescently labeled companion imaging drugs (e.g., CDNP).^[^ ^8^, ^53^ ^]^ Our results show that SAFE probes are entirely compatible with other fluorescent moieties (Figure S6, Supporting Information). For example, neither GFP nor BFP signal from MerTK‐GFP or MC38‐BFP was affected by tetrazine scissors (HK‐Tz) over several cycles. The advantage of SAFE‐IVM is thus that it can be used for serial multiplexing in vivo where a variety of cells can be profiled using commercially available antibodies labeled with C_2_TCO‐linked fluorophores and reference cells can be tracked with other fluorescent moieties.

### 3.4 Future Opportunities and Applications

The above research presents the first evidence that it is feasible to perform cyclic imaging in live mice. In order to expand this concept, we envision a number of future opportunities. For SAFE‐IVM, its multiplexing capability is determined by the amount of residual background accumulated from each cycle. It is particularly challenging to work with live tissue that has multiple parameters that contribute to background signal such as tissue autofluorescence, antibody internalization, cellular debris, and incubation temperature. To address these issues, additional improvements to the bioorthogonal architecture that enable both quenching and releasing of fluorophores are needed. As observed in previous studies, BHQ3 will greatly increase quenching efficiency. When it can be comprehensively and immediately released along with the fluorophores, it will allow in vivo cyclic bioorthogonal imaging as effective as has been shown in other studies using fixed cells, frozen tissue, and live single cells.^[^ ^15^, ^17^ ^]^ SAFE‐IVM could then be applied to longitudinal immunoprofiling of microenvironments that require multiple subset identification and allow observation of dynamic cellular interaction and drug responses as they occur.

## 4 Experimental Section

### Materials

Unless otherwise noted, reactions were carried out under an atmosphere of nitrogen or argon in air‐dried glassware with magnetic stirring. Air‐ and/or moisture‐sensitive liquids were transferred via syringe. All reagents were obtained from commercial sources at the highest grade available and used without further purification. Fluorophores were purchased from Click Chemistry Tools or Fluoroprobes. N‐α‐Boc‐N‐*ε*‐Fmoc‐Lysine was purchased from Chem‐Impex. Amino‐dPEG₄‐CO_2_H was obtained from Quanta BioDesign. Dry solvents and coupling reagents were obtained from Sigma Aldrich. C_2_TCO was a generous gift of Dr. Hannes Mikula (TU Wien, Austria). Anhydrous DMSO and DIPEA for microscale reactions were stored over 3 Å molecular sieves. Column chromatography was carried out with Biotage Snap C18 Bio flash cartridges and a Biotage Isolera Four instrument.

### Antibodies

BSA free antibodies (Table S1, Supporting Information) were purchased from established vendors and stored according to manufacturer recommendations. All antibodies were validated with conventional immunofluorescence imaging (secondary antibody staining) on positive cell lines or mouse splenocytes before usage in SAFE‐IVM imaging.

### Synthesis of SAFE Reactants

Figure 2 summarizes the architectural scheme for 1) HK Tz scissors and 2) the antibody‐C_2_TCO‐fluorochrome linker. Tz scissors and C_2_TCO linkers were synthesized as described previously.^[^ ^26^ ^]^ For fluorophore conjugation, 1 equivalent of DAP‐C_2_TCO‐PEG_4_‐CO_2_H was dissolved in dry DMSO (50 µL µmol^−1^ TCO) and 1.2 equivalents of fluorophore–*N*‐hydroxysuccinimide (NHS) ester in 500 µL of dry DMSO and 4 equivalents of DIPEA were added. When LCMS showed full conversion, the mixture was diluted in aqueous ammonium formate buffer (1 mL, 2.5 mm, pH 9.2) and loaded onto a C18 column, followed by preparative reversed phase column chromatography (ammonium formate buffer [2.5 mm, pH 9.2]/MeCN gradient elution).^[^ ^19^ ^]^

### Kinetics of Quenching

Imaging kinetics were measured using A431 cells cultured in a Millicell EZ 8‐Well Glass Slide (Millipore). The A431 cells were stained with 10 µg mL^−1^ of SAFE‐IVM AF647‐labeled anti‐EGFR antibody (Cetuximab, Selleck Chemicals, A2000) for 10 min at RT. The stained cells were first imaged before addition of HK‐Tz. After the initial round of imaging, 40 µm of HK‐Tz was added to the cells for different lengths of time (5 s–30 min) to measure kinetics. After incubation, cells were washed three times with PBS and imaged.

### Cell Viability

Cell viability was measured using A431 cells cultured in a 96 well plate (Corning, #3362). The cells were treated with different concentrations of HK‐Tz (1.25–160 µm) for 1 h at 37 °C. Following incubation, the cells were washed three times with pre‐warmed media and cell viability was measured 24 h later. PrestoBlue Cell Viability Reagent (Thermo Fisher, A13262) and a TECAN Spark plate reader were used to measure cell viability.

### Antibody Modification with SAFE‐IVM Linkers

Antibodies were first exchanged from their storage solution into PBS‐bicarbonate buffer (PBS supplemented with 100 mm sodium bicarbonate, pH 8.4) using a 40k Zeba Spin column (Thermo Fisher, 87765) for functionalization with fluorophore‐C_2_TCO SAFE probes. After buffer exchange, antibodies were incubated with a four‐ to eight‐fold molar excess of the activated Dye‐C_2_TCO–NHS linker for 25 min at room temperature. The completed conjugation reaction was loaded onto a 40k Zeba column (equilibrated with PBS) for desalting and removal of unreacted dye molecules. The absorbance spectrum of the conjugated antibody was measured using a Nanodrop 1000 (Thermo Scientific) to determine the degree of labeling, using the known extinction coefficients of the dye, IgG antibody, and correction factor (CF280) for the dye absorbance at 280 nm. The SAFE‐modified antibodies were stored in the dark at 4 °C in PBS until usage.

### CDNP Synthesis and Characterization

The synthesis of CDNPs was adapted from previously reported methods.^[^ ^36^, ^54^ ^]^ Briefly, succinyl‐𝛽‐cyclodextrin (Sigma; 250 mg, 1.0 eq. to carboxylate) was dissolved in MES buffer (6 mL, 50 mm, pH = 5) and activated with *N*‐(3‐(dimethylamino)propyl)‐*N*′‐ethylcarbodiimide hydrochloride (Fisher; 1.5 g, 10.0 eq. to carboxylate) and NHS (Sigma; 550 mg, 5.0 eq. to carboxylate) for 30 min at 25 °C in an open scintillation vial (20 mL). A solution containing *L*‐lysine (Sigma; 69.8 mg, 0.5 eq. to carboxylate) in MES buffer (1.5 mL) was added in a drop‐wise manner and the reaction was allowed to stir for 18 h. Under vigorous stirring, saturated brine solution (300 µL) was added to the solution containing the nanoparticles followed by careful addition of ice‐cold absolute ethanol (70 mL), yielding a white precipitate that was collected by centrifugation (1000 × *g*, 1 min). The pellet was resuspended in water (7 mL), filtered through a 0.22 µm centrifugal filter (VWR), and purified with 10 kDa MWCO centrifugal filters (Amicon; 10 000 × *g* for 10 min). The clear syrup‐like solution was washed with water (×3), filtered, and collected. The CDNPs were sterilized by filtration through a 0.22 µm sterile filter (VWR) and lyophilized for 48 h to yield 344 mg of a white powder. The CDNPs were stored at −20 °C until further use. For Pacific Blue‐CDNP synthesis, lyophilized CDNPs (20 mg) were dissolved in carbonate buffer (1 mL, 0.1 m, pH = 8.5) and Pacific Blue Succinimidyl Ester (Fisher; 5 mg mL^−1^ in dimethylformamide) was added to achieve a final concentration of 50 µm. The reaction was stirred for 2 h. The nanoparticles were purified by buffer exchange into water against 10 kDa MWCO centrifugal filters (Amicon; 10 000 × g for 10 min). The filtrate was washed with water (3×) and the final product (Pacific Blue‐CDNPs) was diluted with water to a final concentration of 50 mg mL^−1^. The final product was sterilized by filtration through 0.22 µm sterile filter (VWR). The solution was stored at −20 °C until further use. Particle size and surface charge for CDNP and Pacific Blue‐CDNPs samples were determined by dynamic light scattering and zeta potential, respectively, (Malvern, Zetasizer APS) at a concentration of 5 mg mL^−1^ in PBS buffer (0.1×) (Figure S7, Supporting Information).

### In Vivo Cycling

Mouse cells and tissue inside the DWC were stained with 10 µg mL^−1^ of SAFE‐modified antibodies and incubated for ≈5 min. The staining was indistinguishable with/without blocking with 0.5% BSA‐PBS, so no blocking was used beyond initial feasibility experiments. Following staining, the tissues were washed 3× with PBS containing 100 µm Trolox (Vector labs, CB‐1000). Trolox, a water soluble vitamin E analog antioxidant, was used to minimize any C_2_TCO degradation that might theoretically occur in the presence of reactive oxygen species. Following imaging, a 40 µm solution of HK‐Tz scissors was applied to the tissues. The Tz‐scissors/PBS was incubated for 20 min and then washed three times before the next round. After scission, the staining, imaging, and scission cycle was repeated for multiplexed protein profiling of the same cells. The following permanent stains were used in the last cycle: Calcein AM (Thermo Fisher, C1430) and SYTOX Red (Thermo Fisher, S34859) were used for live and dead cell staining, Hoechst 33342 (Thermo Fisher, H3570) was used for nuclear staining, and tetramethylrhodamine isothiocyanate–dextran (TMRI, MW = 500 000) was used as a vascular stain.

### Imaging and Analysis

Mice were anesthetized using 1.5 L min^−1^ oxygen and 1–3 L min^−1^ isoflurane and stationed on warming plates to avoid hypothermia. DWC live tissues were imaged using a custom‐made Olympus FV1000 confocal multiphoton system using a XLUMPLFLN20xW NA 1.0 water immersion objective. Fluorescent channels were imaged sequentially with 405, 473, 559, and/or 635 nm lasers using a 405/473/559/635 dichroic to separate excitation light and dichroic beam splitters SDM473, SDM560, and SDM640 to separate emission light. Fluorophore signals were further separated using emission filters BA430‐455, BA490‐540, BA575‐620, and BA655‐755. Each fluorescence channel was imaged sequentially using distinct excitation and emission filter sets to ensure minimal bleed‐through between channels. All lasers, beam splitters, and emission filters were purchased from Olympus. For antibody validation, a DeltaVision (Olympus) inverted fluorescence microscope was used to acquire fluorescent images. FITC, Cy3, and Cy5 filter cubes were used to excite MB488, AF555, and AF647 fluorophores, respectively. ImageJ was used for image data analysis.

### Tumor Window Model

Animal studies were conducted in compliance with the National Institutes of Health guide for the care and use of Laboratory animals using female C57BL/6 mice (Jackson, 000664, 6–8 weeks of age). Protocols were approved by the Institutional Animal Care and Use Committees at Massachusetts General Hospital. DWCs were implanted, the upper epidermal layer inside the DWCs removed, and 10^6^ MC38 H2B‐mTagBFP2 tumor cells in 20 µL saline were injected. The brain window chamber was created similarly by removing the calvarium and injecting 10^5^ CT2A‐H2B‐apple GBM cells. The openings were closed with a cover slip and tumor cells allowed to grow and to induce vascularization for at least 8 days before imaging. Serial imaging was performed on both models.

### Intravital Imaging

Images were acquired on an FV1000MPE confocal imaging system (Olympus). Pacific Blue, GFP/YFP, mApple, and VivoTag 680 (VT680) were excited sequentially using 405, 473, 559, and 635 nm diode lasers and BA430‐455, BA490‐540, BA575‐620, and BA655‐755 emission filters with SDM473, SDM560, and SDM640 beam splitters. Images were pseudo‐colored and processed in FIJI (ImageJ, NIH) by adjusting brightness/contrast, creating z‐projections of image stacks and performing a rolling ball background subtraction. To quantify IL‐12^hi^ cell populations, the mApple channel was subtracted from YFP, automated thresholding was applied by the RenyiEntropy method, and ROIs were automatically generated for the corresponding masked image. Cell densities were normalized to pre‐treatment conditions.

### Statistical Analysis

Statistical analyses and line fitting were performed in GraphPad Prism 8. Data was used “as is” and was not pre‐processed. Data were presented as mean ± standard deviation for in vitro studies and as mean ± standard error for in vivo studies. Sample size was included in experimental description and figure legends (*n* = 47 separate mouse imaging experiments). For comparison of two groups, two tailed Student's *t*‐test was used. For multiple comparisons, statistical significance was determined by one‐way ANOVA with post hoc Tukey's HSD test. For temporal data, outliers were identified by Grubb's test and excluded. Comparison was performed by two‐way repeated measures ANOVA with post hoc Fisher's LSD or, for groups of unequal size, by Friedman's Test with post hoc Dunn's test. Significance was assigned at *p* < 0.05.

## Conflict of Interest

The authors declare the filing of a patent which was assigned to Massachusetts General Hospital. The following disclosures are not related to the subject matter of this work. R.W. is a consultant to ModeRNA, Tarveda Therapeutics, Lumicell, Seer, Earli, Aikili Biosystems, and Accure Health.

## Author Contributions

Design: J.K., R.W.; Synthesis: J.C.T.C., E.A.H., M.W.; Experiments: J.K., R.K., K.L.; Data analysis: all authors; Writing: R.W. and all others

## Supporting information

Supporting Information

Supplemental Movie 1

Supplemental Movie 2

Supplemental Movie 3

## Data Availability

The data that support the findings of this study are available from the corresponding author upon reasonable request.
