# Supplementary material for: In Vivo Click Chemistry Enables Multiplexed Intravital Microscopy
Source: Adv Sci (Weinh). 2022 Jun 24;9(24):2200064. doi: 10.1002/advs.202200064 (PMC9405492; doi:10.1002/advs.202200064)
Supplement: Supplementary file 1 — Supporting Information [file ADVS-9-2200064-s002.pdf]

ADVANCED SCIENCE
Open Access

Supporting Information

for *Adv. Sci.*, DOI 10.1002/advs.202200064

In Vivo Click Chemistry Enables Multiplexed Intravital Microscopy

*Jina Ko, Kilean Lucas, Rainer Kohler, Elias A. Halabi, Martin Wilkovitsch, Jonathan C. T. Carlson and Ralph Weissleder**

SUPPLEMENTAL INFORMATION

In vivo click chemistry enables multiplexed intravital microscopy

Jina Ko[1], Kilean Lucas[1], Rainer Kohler[1], Martin Wilkovitsch[2], Elias Halabi[1], Jonathan C. T. Carlson[1,3], Ralph Weissleder[1,4]*

[1] Center for Systems Biology, Massachusetts General Hospital, 185 Cambridge St, CPZN 5206, Boston, MA 02114
[2] Institute of Applied Synthetic Chemistry, TU Wien, 1060 Vienna, Austria
[3] Department of Medicine, Massachusetts General Hospital, Harvard Medical School, Boston, MA 02114, USA
[4] Department of Systems Biology, Harvard Medical School, 200 Longwood Ave, Boston, MA 02115

*R. Weissleder, MD, PhD

Center for Systems Biology

Massachusetts General Hospital

185 Cambridge St, CPZN 5206

Boston, MA, 02114

617-726-8226

rweissleder@mgh.harvard.edu

Figure S1. Unstaining across multiple color channels. Immune cells in dorsal window chambers of live mice were stained with anti-CD45 SAFE-IVM antibodies bearing three different fluorochromes: MB488, AF555, and AF647. The goal of the experiment was to determine i) the co-localization of differently labeled antibodies (i.e. does the fluorochrome affect labeling) and ii) unstaining ability in the three different channels 20 minutes following addition of Tz-scissors. Note the good co-registration and un-staining in the three different channels.

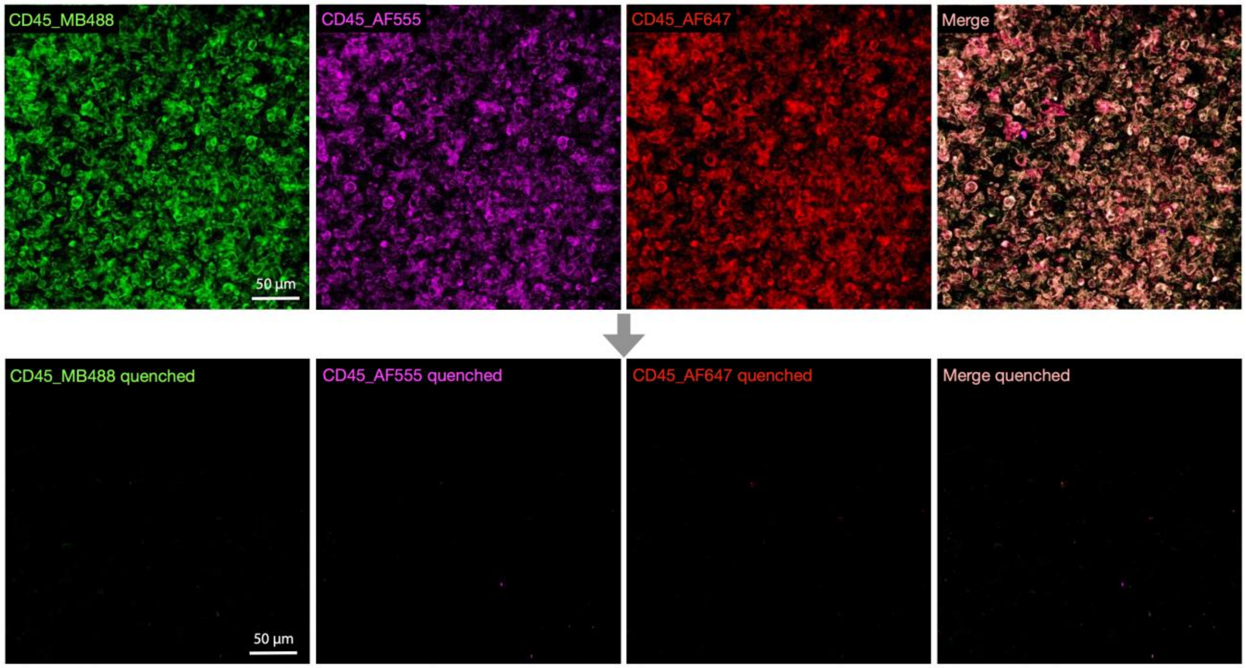

Figure S2. Temporal imaging of SAFE-IVM. MC38-BFP mouse tumor cells (blue) were pre-stained with a non-quenchable anti-MHCII antibody (green) as a control. The cells were imaged *in vivo* every minute during an entire cycling step. SAFE-IVM anti-CD11b (red) was added (T = 8 min) to stain TAM and HK-Tz was subsequently added (T = 15 min) for scission. The SAFE-IVM stained signal (red) disappeared while leaving the fluorescent protein signal (blue) and the green signal intact (n = 3 biological replicates).

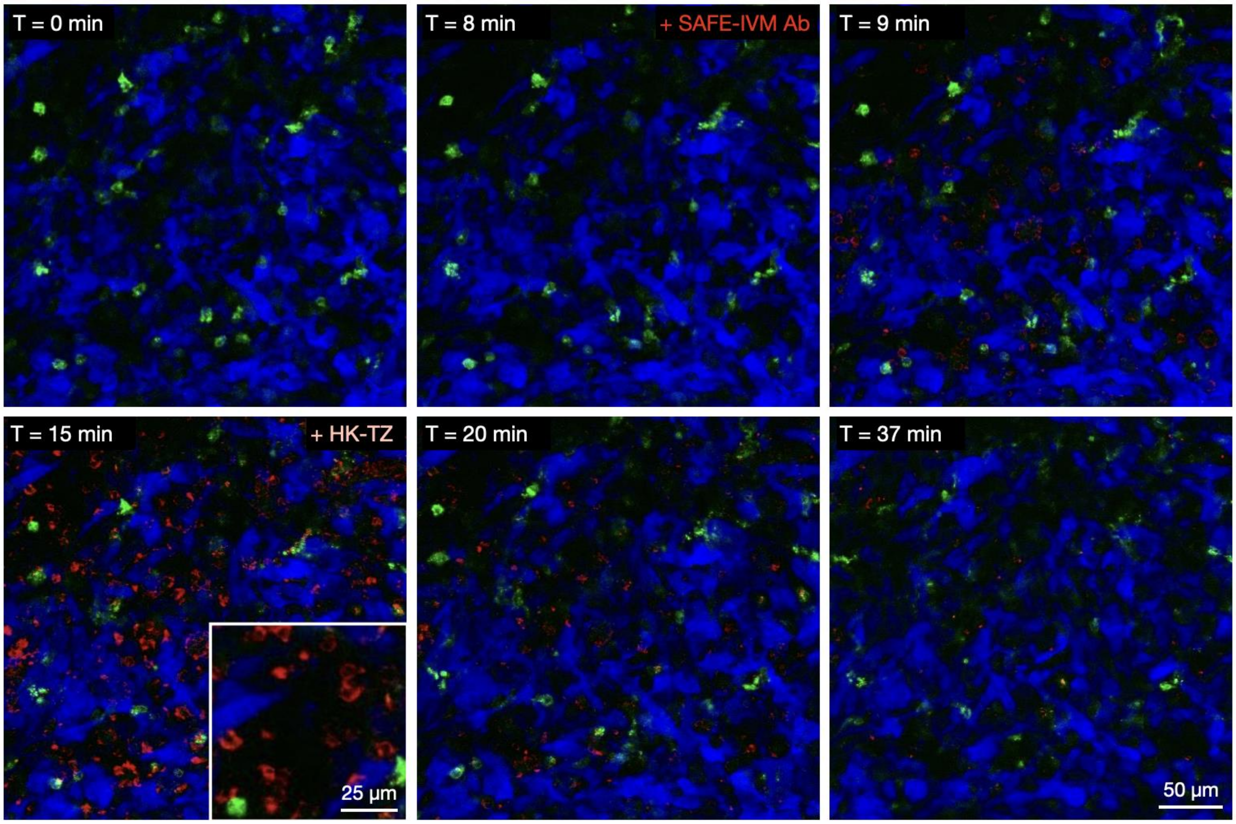

Figure S3. SAFE-IVM antibody testing in mouse splenocytes. Fixed mouse splenocytes were used to validate different antibodies used in this study. They were tested using using MB488, AF555, and AF647 SAFE modified antibodies for their specific staining. The cells showed typical surface staining as would be expected. There was excellent correlation between SAFE imaging and correlative flow cytometry using 12 markers in mouse peripheral blood mononuclear cells (R^2 = 0.987; p <0.0001).

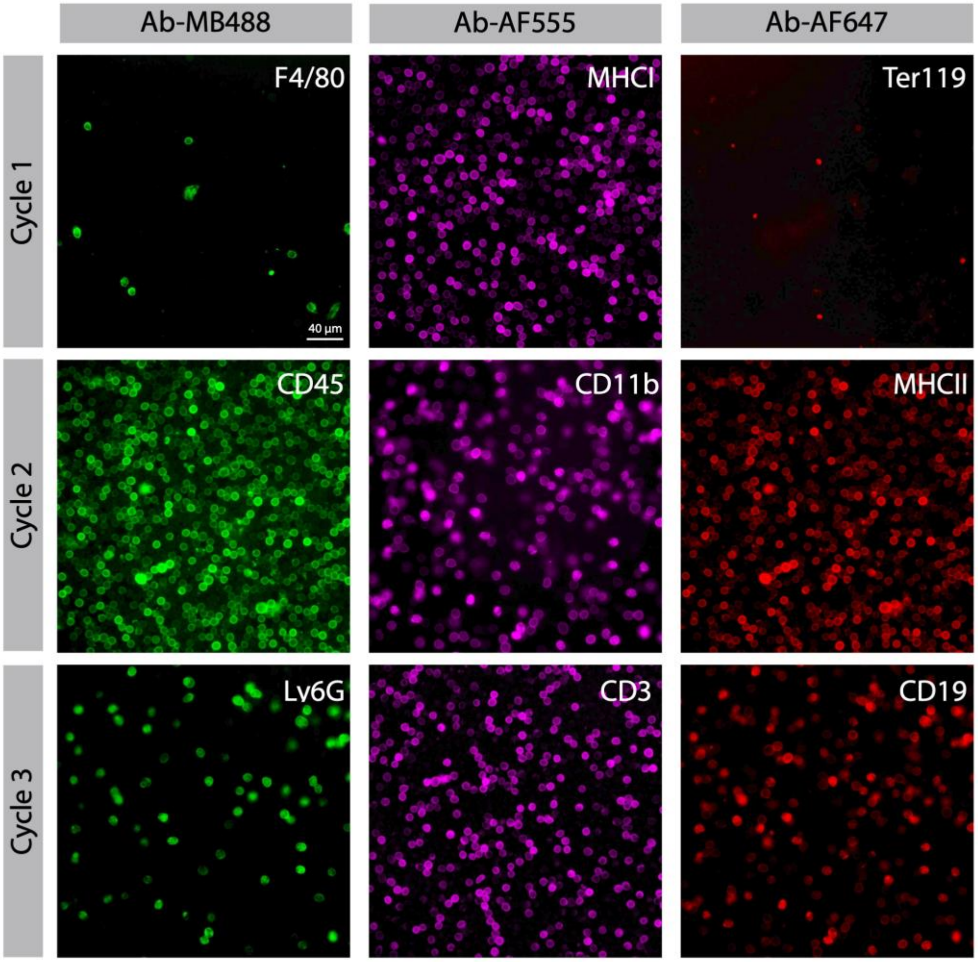

Figure S4. SAFE-IVM steps and imaging time. Each SAFE-IVM cycle takes 35-45 mins, which includes staining (5min), washing (1min), imaging (equilibrium; 5-120min based on applications), scission (20min), and repeat washing (1 min). Imaging can be performed at any time except during the brief wash steps.

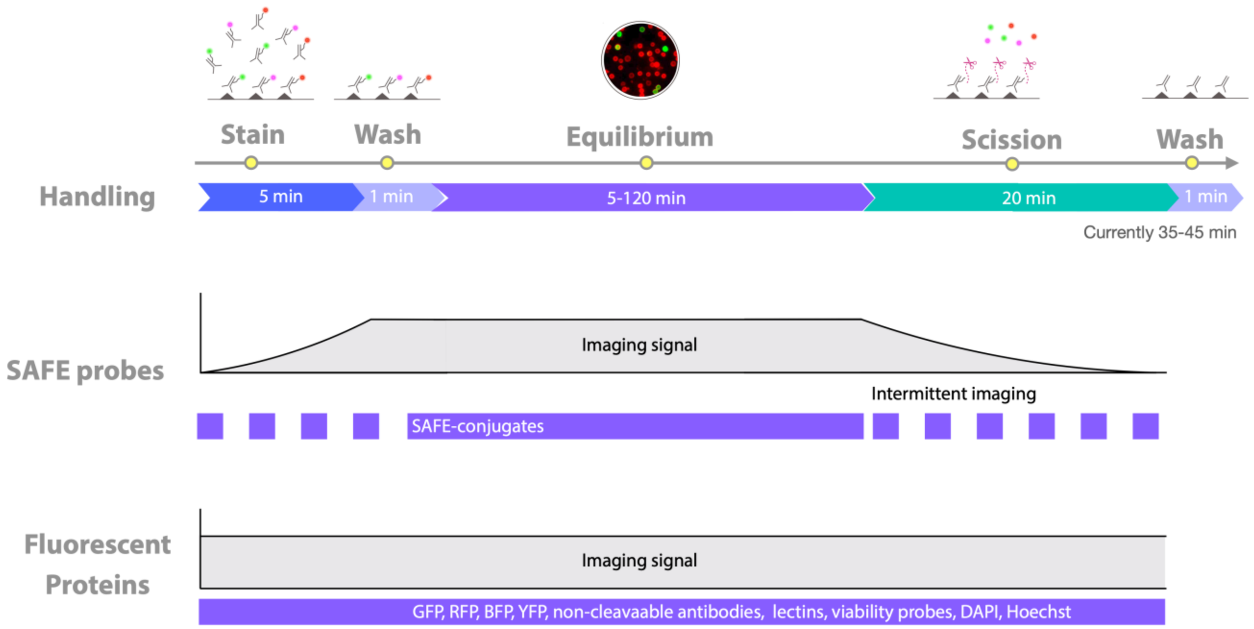

Figure S5. Individual images of CDNP and different immune cell populations from Fig 6. CDNP (white, top right) accumulated in cells 24 hrs after IV administration as shown by punctate clustered signal. Eighty six percent of CDNP associated cells were triple positive for CD11b (red), CD45 (cyan), and MerTk (green), i.e. myeloid derived tumor associated

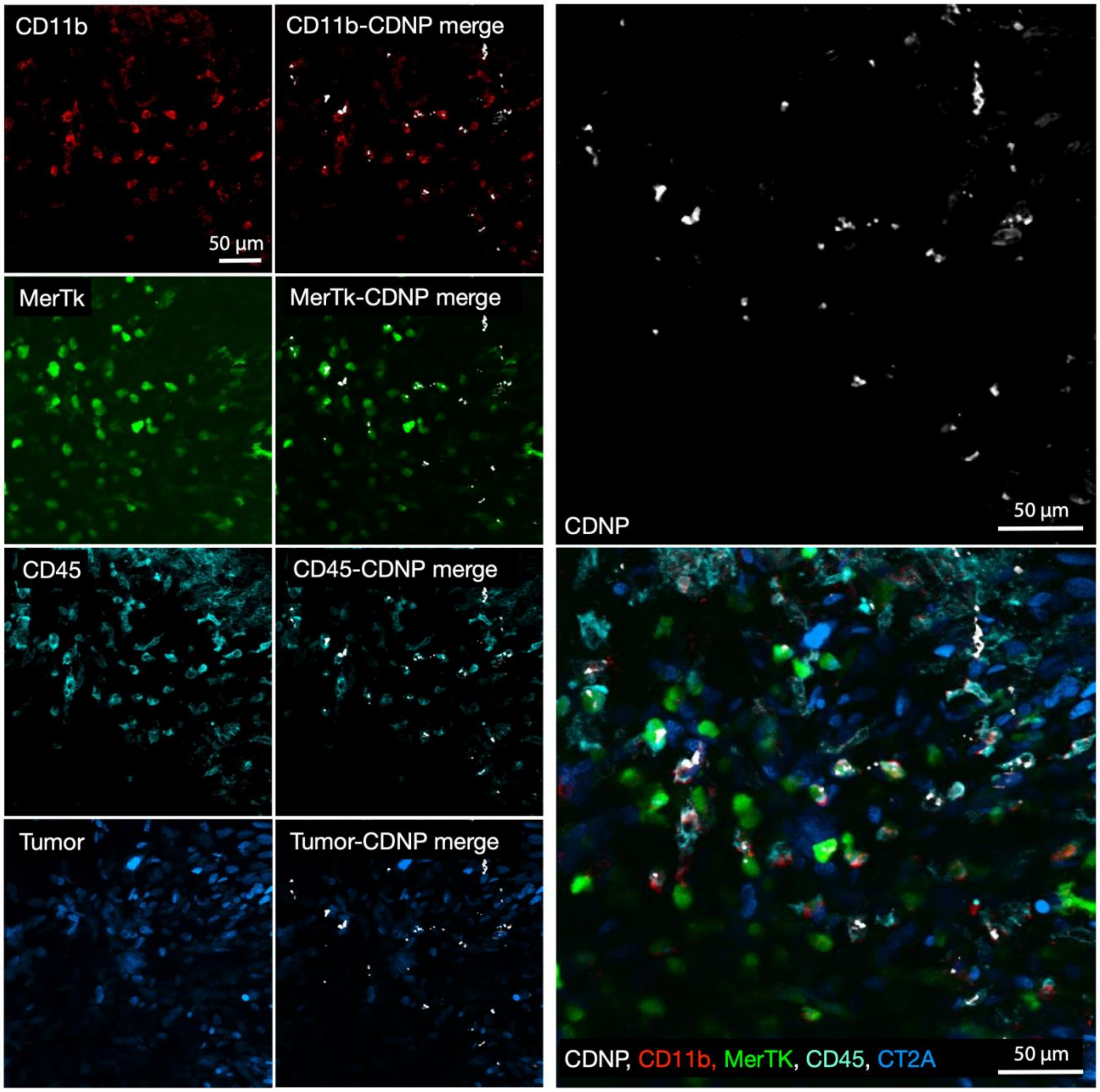

macrophages.

Figure S6. SAFE-IVM probes do not affect fluorescent protein signal. Quantitation of MC38-BFP and MerTK-GFP signal from multiple rounds of cycling during which HK-Tz was administered at 40 µM.

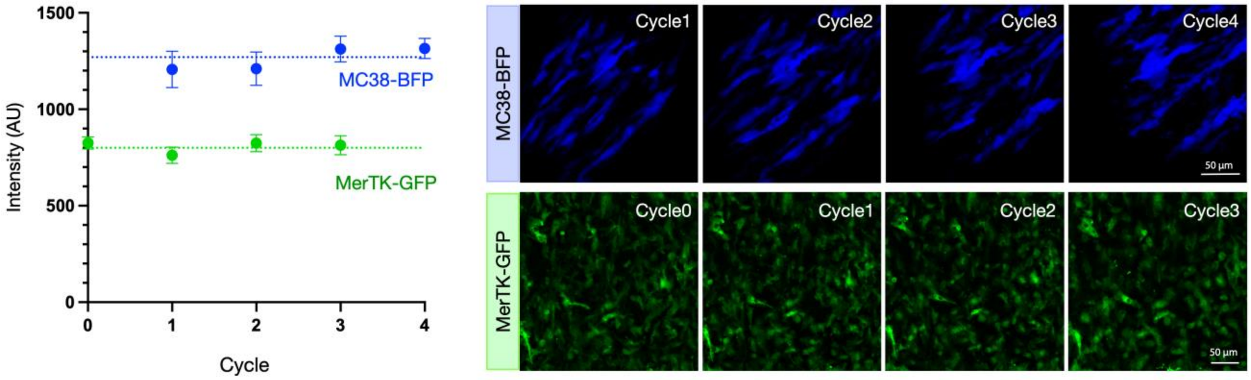

Figure S7. Characterization of CDNP and Pacific Blue-CDNP (PB-CDNP). A. Absorption and emission spectra measured in phosphate buffered normal saline. B. Plots showing the size distribution of the particles determined by DLS measurement. C. Graphs displaying surface charge of the particles determined by zeta potential measurement. All experiments show values from three independent measurements.

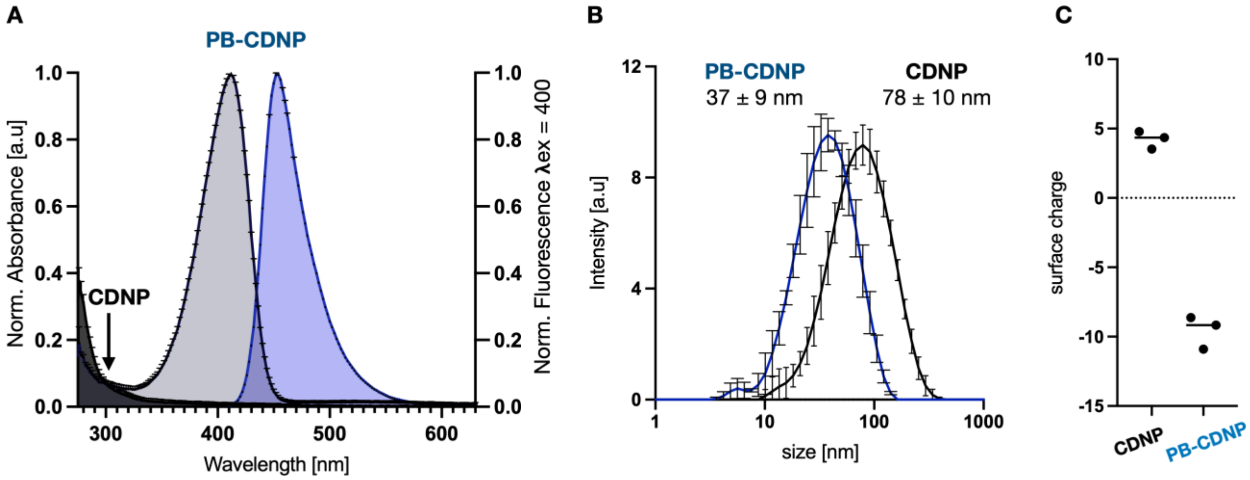

Movie 1. Viability of MerTK-GFP cells in a live mouse after three cycles of Hk-Tz treatment.

Movie 2. Temporal imaging of SAFE-IVM. MC38-BFP mouse tumor cells (blue) were pre-stained with a non-quenchable anti-MHCII antibody (green) as a control. The cells were imaged *in vivo* every minute during an entire cycling step. SAFE-IVM anti-CD11b (red) was added (T = 8 min) to stain TAM and HK-Tz was subsequently added (T = 15 min) for scission. The SAFE-IVM stained signal (red) disappeared while leaving the fluorescent protein signal (blue) and the green signal intact (n = 3). Note the motility of some green cells during the entire cycle process.

Movie 3. Z-stack imaging of MC38-BFP tumors where CD11b (green) and MHCI (red) are stained during an imaging cycle prior to subsequent quenching. The imaging depth is from 0-50 µm.

Table S1: SAFE-IVM antibodies

Target protein	Clone	Host species	Vendor	Catalog #	Degree of labeling	Fluorophore
CD45	104.2	Mouse	Bio X Cell	BE0300	2-5	MB488
						AF555
						AF647
CD11b	M1/70	Rat	Bio X Cell	BE0007	2-4	MB488
						AF647
Ly6G	1A8	Rat	Bio X Cell	BE0075	4.6	MB488
F4/80	QA17A29	Mouse	Biolegend	157301	3.3	AF555
MHCI	M1/42.3.9.8	Rat	Bio X Cell	BE0077	3.6	AF647
MHCII	M5/114	Rat	Bio X Cell	BE0108	3.3	AF555
CD9	MZ3	Rat	Biolegend	124802	2.8	AF555
CD19	1D3	Rat	Bio X Cell	BE0150	4.2	MB488
MHCI	M1/42.3.9.8	Rat	Bio X Cell	BE0077	3.6	AF647
Ter119	TER-119	Rat	Bio X Cell	BE0183	4.7	AF647
